# Generating the Evidence for Typhoid Vaccine Introduction: Considerations for Global Disease Burden Estimates and Vaccine Testing Through Human Challenge

**DOI:** 10.1093/cid/ciz630

**Published:** 2019-10-15

**Authors:** James E Meiring, Alberto Giubilini, Julian Savulescu, Virginia E Pitzer, Andrew J Pollard

**Affiliations:** 1 Oxford Vaccine Group, Department of Paediatrics, University of Oxford, United Kingdom; 2 National Institute for Health Research Oxford Biomedical Research Centre, University of Oxford, United Kingdom; 3 Oxford Uehiro Centre for Practical Ethics, University of Oxford, United Kingdom; 4 Department of Epidemiology of Microbial Diseases, Yale School of Public Health, Yale University, New Haven, Connecticut

**Keywords:** typhoid, vaccines, human challenge, epidemiology, infectious diseases

## Abstract

Typhoid fever has had a major impact on human populations, with the causative pathogen *Salmonella enterica* serovar Typhi implicated in many outbreaks through history. The current burden of disease is estimated at 11–18 million infections annually, with the majority of infections located in Africa and South Asia. Data that have been used to estimate burden are limited to a small number of blood-culture surveillance studies, largely from densely populated urban centers. Extrapolating these data to estimate disease burden within and across countries highlights the lack of precision in global figures. A number of approaches have been developed, characterizing different geographical areas by water-based risk factors for typhoid infection or broader measures of health and development to more accurately extrapolate incidence. Recognition of the substantial disease burden is essential for policy-makers considering vaccine introduction. Typhoid vaccines have been in development for >100 years. The Vi polysaccharide (ViPS) and Ty21a vaccines have had a World Health Organization (WHO) recommendation for programmatic use in countries with high burden for 10 years, with 1 ViPS vaccine also having WHO prequalification. Despite this, uptake and introduction of these vaccines has been minimal. The development of a controlled human infection model (CHIM) enabled the accelerated testing of the newly WHO-prequalified ViPS–tetanus toxoid protein conjugate vaccine, providing efficacy estimates for the vaccine, prior to larger field trials. There is an urgency to the global control of enteric fever due to the escalating problem of antimicrobial resistance. With more accurate burden of disease estimates and a vaccine showing efficacy in CHIM, that control is now a possibility.

Typhoid fever, caused by the ingestion and subsequent mucosal invasion of the pathogen *Salmonella enterica* serovar Typhi (*S.* Typhi), is a serious public health concern in many low- and middle-income countries (LMICs), with an estimated global burden of 11–18 million infections annually [1–3]. In addition, the pathogens *Salmonella enterica* serovars Paratyphi A, B, and C (*S*. Paratyphi), which have a similar clinical presentation to that of *S*. Typhi, are responsible for an estimated 3.4–5.4 million infections globally per year [3, 4].

While improvements in drinking water quality and sanitation have all but eradicated the disease from the majority of developed countries [5–7], short- to medium-term control of the pathogen through vaccination is widely accepted as the best strategy for reducing disease burden in low-income settings [8–10]. Escalating antimicrobial resistance throughout the world, including the 2016–2019 extensively drug-resistant outbreak of *S*. Typhi in Pakistan, threatens the gains in typhoid control and creates concern of a return to the preantibiotic era [11, 12].

However, the generation of evidence to support introduction of an efficacious vaccine against *S*. Typhi into the world’s endemic countries has encountered many challenges. With a nonspecific clinical presentation and no reliable point-of-care diagnostic, predicting accurate disease burden—and therefore demonstrating the need for vaccination to prevent morbidity, mortality, and economic burden—has been problematic.

In addition, with a human-restricted pathogen such as *S.* Typhi, determining the efficacy of new vaccines has been slow, requiring large field trials, which are both expensive and time-consuming. To accelerate vaccine testing, a controlled human infection model (CHIM) for both *S*. Typhi and *S.* Paratyphi has been developed in Oxford, United Kingdom [13–15]. This model has enabled the efficacy testing of a number of candidate vaccines [16], including the recently World Health Organization (WHO)–prequalified typhoid Vi polysaccharide protein conjugate vaccine (Vi-TT) [17, 18], providing data used to support the WHO Strategic Advisory Group of Experts on Immunization (SAGE) recommendation in 2017 for use of typhoid conjugate vaccines (TCVs) in high-burden countries and the Gavi commitment for funding the introduction of TCVs into eligible countries [19, 20].

In this article, we discuss some of the necessary components for the introduction of typhoid vaccines. We bring together the latest approaches for predicting typhoid burden and vaccine impact with a summary of past and current enteric fever vaccines in development, including the ethical requirements surrounding the CHIM used to enable typhoid vaccine efficacy trials.

## Predicting Typhoid Burden and Vaccine Impact

The burden of typhoid fever—that is, the incidence of disease, the severity of illness, the costs to individuals and health systems to treat it, and the extent of antimicrobial resistance—is a key element underlying the need for TCVs and decisions about how best to deploy them. However, the burden of typhoid fever is difficult to quantify. The symptoms of the disease, including prolonged fever and abdominal discomfort, are nonspecific and easily confused with other potential diagnoses [21]. Confirmation of typhoid cases currently relies upon blood culture, which is difficult to implement in many low-income settings and suffers from poor sensitivity [22].

Typhoid incidence was directly assessed in only 22 sites in 14 different countries between 1980 and 2017, with the recent Typhoid Surveillance in Africa Program adding additional data from 13 sites across 10 African countries [1, 23, 24]. The limited geographical breadth of burden data presents challenges when trying to extrapolate from data for just a few sites to estimate the global incidence of typhoid fever. A variety of approaches have been used to estimate the incidence of typhoid fever from the available data. Some models have estimated the incidence by region and assumed that incidence is similar in nearby regions without data [2, 4, 25], with or without adjusting for typhoid risk factors, including the percentage of the population living in urban slums and rural locations without access to improved water [2]. Other studies used the available data to identify the best predictors of typhoid fever incidence from a variety of possible covariates [1, 3]. One such study found that general measures of health (eg, prevalence of stunting) and development (eg, percentage of roads paved, percentage of the population living in extreme poverty) were more predictive of typhoid fever incidence than widely used measures of access to improved water and sanitation [1]. As a result of the different methods used, estimates of typhoid incidence have shown considerable variation, particularly for countries with few or no data [26]. Furthermore, typhoid incidence has been known to change over time—sometimes dramatically—as evidenced by recent multiyear outbreaks that have been occurring across parts of sub-Saharan Africa associated with the emergence of antimicrobial-resistant strains [27].

For countries without surveillance data, the recently published WHO Surveillance Standards for Vaccine-Preventable Diseases advise that the minimum surveillance for typhoid-endemic countries should be laboratory or facility based [28]. This could be either the routine passive reporting of positive laboratory results for invasive *Salmonella* to a surveillance system, or an active approach where patients meeting set criteria are identified in a number of sentinel facilities for blood culture collection. While this method of surveillance would not identify all the cases of enteric fever within a population, it would allow disease patterns to be monitored over time, both pre- and postvaccine introduction. This approach has been well demonstrated from sentinel surveillance sites in some LMICs [29]. It could also be integrated with other disease reporting systems already operating within the country and would allow the reporting of antimicrobial susceptibility data. It would also be of added benefit to choose surveillance sites from different contexts within the country (eg, urban vs rural).

Published data from Bangladesh have provided an example of how this might be achieved [30]. Using the Global Invasive Bacterial Vaccine-Preventable Diseases Surveillance Network designed for pathogens such as *Streptococcus pneumoniae* and *Haemophilus influenzae* type b, clinicians expanded the inclusion criteria for blood culture collection in children admitted to sentinel hospital facilities to perform typhoid surveillance. This resulted in a 25% increase in number of blood cultures performed, but a 5-fold increase in detection of enteric fever cases. This was introduced with only a modest increase in costs of US$44 974 annually. For countries wanting to establish typhoid fever surveillance and to monitor impact of vaccine, this may provide a platform that is both affordable and implementable.

To assess the value of typhoid vaccination strategies, it is also important to know severity and costs associated with typhoid illness. The hospitalization and case fatality rates are particularly influential when estimating the cost-effectiveness of typhoid vaccination strategies [31–33]. However, studies have found considerable heterogeneity in the hospitalization and case fatality rates of typhoid fever cases in different parts of the world [34, 35]. In particular, estimates of the case fatality rate that are derived only from blood culture–confirmed patients may overestimate the mortality rate, as these cases could represent the more severe end of the spectrum of illness. Conversely, typhoid cases with intestinal perforations tend to exhibit higher case fatality rates [36], but fatal intestinal perforation cases may have lower rates of blood culture confirmation. Estimates of the costs of treating typhoid fever are also highly variable [37], and few studies have measured the costs to individual patients themselves. The impact of antimicrobial resistance should also be factored into these cost estimates.

TCVs are likely to provide protection not only for those who receive the vaccine, but also indirect protection (ie, herd immunity) by reducing typhoid transmission [38]. Mathematical models of typhoid transmission have been developed to predict the population-level impact of different vaccination strategies against typhoid fever [31, 32, 39]. These models generally predict that the benefits of a population-wide vaccination strategy will exceed the reduction in incidence expected among vaccinated individuals alone. However, the level of indirect protection is difficult to predict and will depend on the ability of TCVs to prevent not only typhoid fever, but also shedding of the bacteria from subclinical cases [38]. Mathematical models attempt to infer the prevalence of infection from the observed cases of disease and understanding of the natural history of the pathogen, but are difficult to validate. The prevalence of chronic carriers and the role they play in transmission will influence the predicted level of indirect protection from vaccination [39, 40].

## Enteric Fever Vaccines: Development and Clinical Testing

Development of enteric fever vaccines started in the late 19th century with the first heat-killed vaccines (see also Gradmann et al., S385 in this supplement) [41]. In the 1950–1960s, whole cell vaccines (WCVs) went through a number of efficacy trials, with a meta-analysis suggesting a cumulative 3-year efficacy of 73% [42] with introduction of 1 WCV into the Expanded Programme on Immunization in Thailand [43]. Unfortunately, this vaccine caused high rates of systemic and localized side effects, leading to school absenteeism in children [44].

In the 1970–1980s, 2 additional vaccines were developed. Ty21a is an oral live attenuated vaccine that went on to large-scale field trials after showing promising efficacy results (87% after 5–8 doses) in a CHIM in Maryland (see also Kirchhelle et al, S388 in this supplement) [45]. Meta-analysis of these trials demonstrated a cumulative efficacy of 50% at 3 years [42, 46]. Analysis from these trials also demonstrated an estimated protective efficacy against *S.* Paratyphi B of 45% (95% confidence interval [CI], 8%–73%) [47].

Vi polysaccharide (ViPS) is a parenteral vaccine containing the purified capsular ViPS antigen. Combining results from individually and cluster randomized trials, efficacy at 2 years for ViPS was 59% [46]. ViPS is not licensed in children <2 years of age due to poor immunogenicity. Because the vaccine contains a T-cell–independent antigen, it does not induce immunological memory and cannot be boosted with repeated vaccination [48].

However, conjugate vaccines, where the polysaccharide capsule is chemically conjugated to a protein carrier, do produce a T-cell–dependent response [49–51]. This technology has now been applied to both the Vi antigen, with a number of TCVs in various stages of development, and the O-specific polysaccharide from *S.* Paratyphi A. Vaccines in clinical testing are summarized in Table 1.

**Table 1.** Typhoid and Paratyphoid Conjugate Vaccines, Licensed or in Late-stage Clinical Development

Vaccine	Vi-rEPA^a^	Vi-DT	Vi-CRM_197_	Vi-TT (PedaTyph)	Vi-TT (Tybar-TCV)	O:2-TT
Pathogen target	*S.* Typhi	*S.* Typhi	*S.* Typhi	*S.* Typhi	*S.* Typhi	*S.* Paratyphi A
Carrier protein	Recombinant *Pseudomonas aeruginosa* exoprotein A	Diphtheria toxoid	Diphtheria toxin CRM_197_	Tetanus toxoid	Tetanus toxoid	Tetanus toxoid
Clinical trials (n = 14)						
Safety and immunogenicity	18–44 years of age, US [52]; 2–44 years of age, Vietnam [53]	2–45 years of age, Philippines [54]; 2–40 years of age, Indonesia [55]	18–40 years of age, Belgium [56]; 6 weeks to 45 years of age, Pakistan, India, and Philippines [57]	3 months to 5 years of age, India [58, 59]; 6 months to 12 years of age, India [60]	6 months to 45 years of age, India [61]; 18–60 years of age, UK[18]	2–44 years of age, Vietnam [62]
Efficacy	2–5 years of age, Vietnam; protective efficacy 91.5% at 27 months, 89% at 47 months [63, 64]	None	None	6 months to 12 years of age, India; protective efficacy 100%^b^ (95% CI, 97.6%–100%) at 12 months [60]	18–60 years of age, UK; protective efficacy 54.6%–87.1% at 14 days [18]	None
Developer(s)	US NIH; Lanzhou Institute of Biological Products, China	International Vaccine Institute, Korea; SK Bioscience, Korea; Bio Farma, Indonesia	GSK Vaccines for Global Health, Italy; Biological E, India	Bio Med, India	Bharat Biotech, India	US NIH; Changchun Institute of Biological Products, China; Lanzhou Institute of Biological Products, China
Licensed	…	…	…	India	India, Nepal, Pakistan, Zimbabwe, Nigeria, Cambodia	…

Abbreviations: CI, confidence interval; NIH, National Institutes of Health; O:2-TT, O-specific polysaccharide-tetanus toxoid; TCV, typhoid conjugate vaccine; UK, United Kingdom; US, United States; Vi-CRM_197_, Vi-non-toxic variant of diptheria toxin; Vi-DT, Vi-diptheria toxoid; Vi-rEPA, Vi-*Pseudomonas aeruginosa* exoprotein A; Vi-TT, Vi-tetanus toxoid.

^a^This vaccine went through a full clinical development program but is not licensed.

^b^This trial had a small sample size, with <100 children aged <2 years of age receiving TCV; therefore, any firm conclusions are unable to be drawn from it.

## Typhoid CHIM: Enabling Vaccine Testing

The most widely used TCV, Typbar-TCV (Bharat Biotech, India), first licensed for use in India, received WHO prequalification in January 2018 [17], supported by efficacy data from the Oxford CHIM. In this study, 112 healthy participants received either Vi-TT, ViPS, or a control meningococcal vaccine in a 1:1:1 double-blind randomization. One month following vaccination, participants were orally challenged with *S.* Typhi bacteria and were followed up as previously described [13]. From this model, the protective efficacy (PE) of Vi-TT against typhoid infection (defined as persistent fever or bacteremia) was 54.6% (95% CI, 26.8%–71.8%), which was comparable with the PE of ViPS at 52% (95% CI, 23.2%–70.0%). In the model, following oral challenge with the pathogen, participants have blood culture collection daily for the subsequent 14 days regardless of symptoms. In field settings, a participant would only have blood culture collection after the development of fever. Applying this case definition of fever followed by the identification of bacteremia, which may more closely simulate a “real-world” field trial, PE of Vi-TT increased to 87.1% (95% CI, 47.2%–96.9%) compared with 52.3% (−4.2% to 78.2%) for ViPS [18].

TCVs now have a WHO SAGE recommendation for use from 6 months of age in typhoid-endemic regions, with routine introduction to be prioritized in countries with high burden of disease and/or high rates of antimicrobial resistance [19, 48] and commitment from Gavi to fund introduction into eligible countries, with field trials under way providing the data to inform this implementation [8, 20, 65]. There remains a need for vaccines against *S.* Paratyphi A, and the paratyphoid model also developed in Oxford may prove useful in further vaccine testing [14].

For pathogens like *S.* Typhi, the use of CHIM has the potential to accelerate vaccine evaluation and introduction [66]. It has proven to be a valuable tool in this case, among a wider toolkit of additional measures including field trials and immunogenicity data [61]. There are important ethical points that must be considered in the development and use of any CHIM. In challenge studies, participants are intentionally exposed to a risk to benefit scientific research and therefore the risk must be worth taking and sufficiently small. The risk should therefore be known before commencing the study; nonhuman or epidemiological work should be performed to estimate risk and show the necessity of human subjects. In addition, the risk must be proportionate to the expected benefits of the study and “under no circumstances should the research expose volunteers to risks of irreversible, incurable or possibly fatal infections” [67–69]. Participation in the study must be free and autonomous, with reimbursement for time and travel. Participants should understand all the risks involved, the potential benefits, and the options they have for opting out. Successfully applying these conditions to the challenge of humans with *S*. Typhi has enabled the safe and rapid testing of vaccines described in this review.

## CONCLUSIONS

Through the use of mathematical modeling and extrapolation of incidence data across nonsurveyed regions, typhoid burden estimates, including case fatality rates and the impact of antimicrobial resistance, are becoming more robust. Combining these data with cost-effectiveness estimates, the case for vaccine introduction into endemic countries is stronger. The development and use of CHIMs to accelerate the testing of vaccines has, as with other pathogens, proven to be a valuable addition to large-scale field trials. With the availability and funding of efficacious typhoid vaccines and the continued development of vaccines for paratyphoid, the global control of enteric fever could now be significantly strengthened.

## Notes

***Financial support.*** This work was supported by the Bill & Melinda Gates Foundation (grant numbers OPP1116967 and OPP1151153) and the Wellcome Trust (strategic award 106158/Z/14/Z).

***Supplement sponsorship.*** This supplement has been funded by the New Venture Fund.

***Potential conflicts of interest.*** A. J. P. is Chair of the UK Department of Health and Social Care’s Joint Committee on Vaccination and Immunisation and the European Medicines Agency scientific advisory group on vaccines and is a member of the World Health Organization’s (WHO) Strategic Advisory Group of Experts on Immunization. A. J. P. reports funding from the National Institute for Health Research Oxford Biomedical Research Centre. V. E. P. has received reimbursement from Merck for travel expenses unrelated to the subject of this manuscript and is a member of the WHO Immunization and Vaccine-Related Implementation Research Advisory Committee. All other authors report no potential conflicts of interest. All authors have submitted the ICMJE Form for Disclosure of Potential Conflicts of Interest. Conflicts that the editors consider relevant to the content of the manuscript have been disclosed.

## References

[1] Antillón M, Warren JL, Crawford FW, et al The burden of typhoid fever in low- and middle-income countries: a meta-regression approach. PLoS Negl Trop Dis 2017; 11:e0005376. 28241011 10.1371/journal.pntd.0005376 PMC5344533

[2] Mogasale V, Maskery B, Ochiai RL, et al. Burden of typhoid fever in low-income and middle-income countries: a systematic, literature-based update with risk-factor adjustment. Lancet Glob Health 2014; 2:e570–80. 25304633 10.1016/S2214-109X(14)70301-8

[3] GBD 2017 Typhoid and Paratyphoid Collaborators. The global burden of typhoid and paratyphoid fevers: a systematic analysis for the Global Burden of Disease Study 2017. Lancet Infect Dis 2019; 19:369–81. 30792131 10.1016/S1473-3099(18)30685-6 PMC6437314

[4] Crump JA, Luby SP, Mintz ED The global burden of typhoid fever. Bull World Health Organ 2004; 82:346–53. 15298225 PMC2622843

[5] Sedgwick WT, Macnutt JS On the Mills-Reincke phenomenon and Hazen s theorem concerning the decrease in mortality from diseases other than typhoid fever following the purification of public water-supplies. J Infect Dis 1910; 7:489–564.

[6] Cutler D, Miller G The role of public health improvements in health advances: the twentieth-century United States. Demography 2005; 42:1–22. 15782893 10.1353/dem.2005.0002

[7] UN-Water. WHO/UNICEF Joint Monitoring Programme for Water Supply and Sanitation. Available at: https://www.unwater.org/publication_categories/whounicef-joint-monitoring-programme-for-water-supply-sanitation-hygiene-jmp/. Accessed 31 March 2017.

[8] Meiring JE, Gibani M; TyVAC Consortium Meeting Group The Typhoid Vaccine Acceleration Consortium (TyVAC): vaccine effectiveness study designs: accelerating the introduction of typhoid conjugate vaccines and reducing the global burden of enteric fever. Report from a meeting held on 26-27 October 2016, Oxford, UK. Vaccine 2017; 35:5081–8. 28802757 10.1016/j.vaccine.2017.08.001

[9] Bentsi-Enchill AD, Pollard AJ A turning point in typhoid control. J Infect Dis 2018; 218:185–7. 10.1093/infdis/jiy417 PMC6226784 30189009

[10] Gibani MM, Britto C, Pollard AJ Typhoid and paratyphoid fever: a call to action. Curr Opin Infect Dis 2018; 31:440–8. 30138141 10.1097/QCO.0000000000000479 PMC6319573

[11] Klemm EJ, Shakoor S, Page AJ, et al Emergence of an extensively drug-resistant *Salmonella enterica* serovar Typhi clone harboring a promiscuous plasmid encoding resistance to fluoroquinolones and third-generation cephalosporins. MBio 2018; 9:e00105-18. 29463654 10.1128/mBio.00105-18 PMC5821095

[12] Andrews JR, Qamar FN, Charles RC, Ryan ET Extensively drug-resistant typhoid—are conjugate vaccines arriving just in time? N Engl J Med 2018; 379:1493–5. 30332569 10.1056/NEJMp1803926

[13] Waddington CS, Darton TC, Jones C, et al. An outpatient, ambulant-design, controlled human infection model using escalating doses of *Salmonella* Typhi challenge delivered in sodium bicarbonate solution. Clin Infect Dis 2014; 58:1230–40. 24519873 10.1093/cid/ciu078 PMC3982839

[14] Dobinson HC, Gibani MM, Jones C, et al. Evaluation of the clinical and microbiological response to *Salmonella* Paratyphi A infection in the first paratyphoid human challenge model. Clin Infect Dis 2017; 64:1066–73. 28158395 10.1093/cid/cix042 PMC5439345

[15] McCullagh D, Dobinson HC, Darton T, et al. Understanding paratyphoid infection: study protocol for the development of a human model of *Salmonella enterica* serovar Paratyphi A challenge in healthy adult volunteers. BMJ Open 2015; 5:e007481. 10.1136/bmjopen-2014-007481 PMC4480031 26082464

[16] Darton TC, Jones C, Blohmke CJ, et al Using a human challenge model of infection to measure vaccine efficacy: a randomised, controlled trial comparing the typhoid vaccines M01ZH09 with placebo and Ty21a. PLoS Negl Trop Dis 2016; 10:e0004926. 27533046 10.1371/journal.pntd.0004926 PMC4988630

[17] Burki T Typhoid conjugate vaccine gets WHO prequalification. Lancet Infect Dis 2018; 18:258. 29485093 10.1016/S1473-3099(18)30088-4 PMC7129039

[18] Jin C, Gibani MM, Moore M, et al Efficacy and immunogenicity of a Vi-tetanus toxoid conjugate vaccine in the prevention of typhoid fever using a controlled human infection model of *Salmonella* Typhi: a randomised controlled, phase 2b trial. Lancet 2017; 390:2472–80. 28965718 10.1016/S0140-6736(17)32149-9 PMC5720597

[19] World Health Organization SAGE meeting of October 2017. Geneva, Switzerland: WHO, 2017.

[20] Gavi, the Vaccine Alliance Millions of children set to be protected against typhoid fever. Available at: https://www.gavi.org/library/news/press-releases/2017/millions-of-children-set-to-be-protected-against-typhoid-fever/. Accessed 19 April 2018.

[21] Parry CM, Hien TT, Dougan G, White NJ, Farrar JJ Typhoid fever. N Engl J Med 2002; 347:1770–82. 12456854 10.1056/NEJMra020201

[22] Antillon M, Saad NJ, Baker S, Pollard AJ, Pitzer VE The relationship between blood sample volume and diagnostic sensitivity of blood culture for typhoid and paratyphoid fever: a systematic review and meta-analysis. J Infect Dis 2018; 218:255–67. 10.1093/infdis/jiy471 PMC6226661 30307563

[23] Mogasale V, Mogasale VV, Ramani E, et al. Revisiting typhoid fever surveillance in low and middle income countries: lessons from systematic literature review of population-based longitudinal studies. BMC Infect Dis 2016; 16:35. 26822522 10.1186/s12879-016-1351-3 PMC4731936

[24] Marks F, von Kalckreuth V, Aaby P, et al. Incidence of invasive *Salmonella* disease in sub-Saharan Africa: a multicentre population-based surveillance study. Lancet Glob Health 2017; 5:e310–23. 28193398 10.1016/S2214-109X(17)30022-0 PMC5316558

[25] Buckle GC, Walker CL, Black RE Typhoid fever and paratyphoid fever: systematic review to estimate global morbidity and mortality for 2010. J Glob Health 2012; 2:010401. 23198130 10.7189/jogh.02.010401 PMC3484760

[26] Radhakrishnan A, Als D, Mintz ED, et al. Introductory article on global burden and epidemiology of typhoid fever. Am J Trop Med Hyg 2018; 99. 10.4269/ajtmh.18-0032 PMC6128367 30047370

[27] Feasey NA, Gaskell K, Wong V, et al Rapid emergence of multidrug resistant, H58-lineage *Salmonella* Typhi in Blantyre, Malawi. PLoS Negl Trop Dis 2015; 9:e0003748. 25909750 10.1371/journal.pntd.0003748 PMC4409211

[28] World Health Organization Vaccine-preventable diseases surveillance standards. Geneva, Switzerland: WHO, 2018.

[29] Musicha P, Cornick JE, Bar-Zeev N, et al. Trends in antimicrobial resistance in bloodstream infection isolates at a large urban hospital in Malawi (1998-2016): a surveillance study. Lancet Infect Dis 2017; 17:1042–52. 28818544 10.1016/S1473-3099(17)30394-8 PMC5610140

[30] Saha S, Islam M, Uddin MJ, et al. Integration of enteric fever surveillance into the WHO-coordinated Invasive Bacterial-Vaccine Preventable Diseases (IB-VPD) platform: a low cost approach to track an increasingly important disease. PLoS Negl Trop Dis 2017; 11:e0005999. 29073137 10.1371/journal.pntd.0005999 PMC5658195

[31] Antillón M, Bilcke J, Paltiel AD, Pitzer VE Cost-effectiveness analysis of typhoid conjugate vaccines in five endemic low- and middle-income settings. Vaccine 2017; 35:3506–14. 28527687 10.1016/j.vaccine.2017.05.001 PMC5462484

[32] Lo NC, Gupta R, Stanaway JD, et al. Comparison of strategies and incidence thresholds for Vi conjugate vaccines against typhoid fever: a cost-effectiveness modeling study. J Infect Dis 2018; 218:232–42. 10.1093/infdis/jix598 PMC6226717 29444257

[33] Bilcke J, Antillón M, Pieters Z, et al. Cost-effectiveness of routine and campaign use of typhoid Vi-conjugate vaccine in Gavi-eligible countries: a modelling study. Lancet Infect Dis 2019; 19:728–39. 31130329 10.1016/S1473-3099(18)30804-1 PMC6595249

[34] Pieters Z, Saad NJ, Antillón M, Pitzer VE, Bilcke J Case fatality rate of enteric fever in endemic countries: a systematic review and meta-analysis. Clin Infect Dis 2018; 67:628–38. 29522159 10.1093/cid/ciy190 PMC6070077

[35] Lee JS, Mogasale VV, Mogasale V, Lee K Geographical distribution of typhoid risk factors in low and middle income countries. BMC Infect Dis 2016; 16:732. 27919235 10.1186/s12879-016-2074-1 PMC5139008

[36] Mogasale V, Desai SN, Mogasale VV, Park JK, Ochiai RL, Wierzba TF Case fatality rate and length of hospital stay among patients with typhoid intestinal perforation in developing countries: a systematic literature review. PLoS One 2014; 9:e93784. 24743649 10.1371/journal.pone.0093784 PMC3990554

[37] Poulos C, Riewpaiboon A, Stewart JF, et al; DOMI Typhoid COI Study Group Cost of illness due to typhoid fever in five Asian countries. Trop Med Int Health 2011; 16:314–23. 21223462 10.1111/j.1365-3156.2010.02711.x

[38] Gibani MM, Voysey M, Jin C, et al The impact of vaccination and prior exposure on stool shedding of *Salmonella* Typhi and *Salmonella* Paratyphi in 6 controlled human infection studies. Clin Infect Dis 2018; 68:1265–73. 10.1093/cid/ciy670 PMC6452003 30252031

[39] Pitzer VE, Bowles CC, Baker S, et al. Predicting the impact of vaccination on the transmission dynamics of typhoid in South Asia: a mathematical modeling study. PLoS Negl Trop Dis 2014; 8:e2642. 24416466 10.1371/journal.pntd.0002642 PMC3886927

[40] Darton TC, Meiring JE, Tonks S, et al; STRATAA Study Consortium The STRATAA study protocol: a programme to assess the burden of enteric fever in Bangladesh, Malawi and Nepal using prospective population census, passive surveillance, serological studies and healthcare utilisation surveys. BMJ Open 2017; 7:e016283. 10.1136/bmjopen-2017-016283 PMC5726077 28674145

[41] Hejfec LB Results of the study of typhoid vaccines in four controlled field trials in the USSR. Bull World Health Organ 1965; 32:1–14. 14290076 PMC2555188

[42] Engels EA, Falagas ME, Lau J, Bennish ML Typhoid fever vaccines: a meta-analysis of studies on efficacy and toxicity. BMJ 1998; 316:110–6. 9462316 10.1136/bmj.316.7125.110 PMC2665386

[43] Bodhidatta L, Taylor DN, Thisyakorn U, Echeverria P Control of typhoid fever in Bangkok, Thailand, by annual immunization of schoolchildren with parenteral typhoid vaccine. Rev Infect Dis 1987; 9:841–5. 3438648 10.1093/clinids/9.4.841

[44] World Health Organization. Typhoid vaccines: WHO position paper. Wkly Epidemiol Rec 2008; 83:49–59. 18260212

[45] Hornick RB, DuPont HL, Levine MM, et al. Efficacy of a live oral typhoid vaccine in human volunteers. Dev Biol Stand 1976; 33:89–92. 782976

[46] Milligan R, Paul M, Richardson M, Neuberger A Vaccines for preventing typhoid fever. Cochrane Database Syst Rev 2018; 5:CD001261. 29851031 10.1002/14651858.CD001261.pub4 PMC6494485

[47] Levine MM, Ferreccio C, Black RE, Lagos R, San Martin O, Blackwelder WC Ty21a live oral typhoid vaccine and prevention of paratyphoid fever caused by *Salmonella enterica* serovar Paratyphi B. Clin Infect Dis 2007; 45(Suppl 1):S24–8. 17582564 10.1086/518141

[48] World Health Organization. Typhoid vaccines position paper. Geneva, Switzerland: WHO, 2018. 10.1016/j.vaccine.2018.04.022 29661581

[49] Obbins JB, Schneerson R, Porter A, Smith DH Prevention of systemic infections, especially meningitis, caused by *Haemophilus influenzae* type b. JAMA 1996; 276:1181. 8827975 10.1001/jama.276.14.1181

[50] Goldblatt D Conjugate vaccines. Clin Exp Immunol 2000; 119:1–3. 10671089 10.1046/j.1365-2249.2000.01109.x PMC1905528

[51] Pichichero ME Protein carriers of conjugate vaccines: characteristics, development, and clinical trials. Hum Vaccin Immunother 2013; 9:2505–23. 23955057 10.4161/hv.26109 PMC4162048

[52] Szu SC, Taylor DN, Trofa AC, et al. Laboratory and preliminary clinical characterization of Vi capsular polysaccharide-protein conjugate vaccines. Infect Immun 1994; 62:4440–4. 7927707 10.1128/iai.62.10.4440-4444.1994 PMC303128

[53] Kossaczka Z, Lin FY, Ho VA, et al. Safety and immunogenicity of Vi conjugate vaccines for typhoid fever in adults, teenagers, and 2- to 4-year-old children in Vietnam. Infect Immun 1999; 67:5806–10. 10531232 10.1128/iai.67.11.5806-5810.1999 PMC96958

[54] Capeding MR, Teshome S, Saluja T, et al. Safety and immunogenicity of a Vi-DT typhoid conjugate vaccine: phase I trial in Healthy Filipino adults and children. Vaccine 2018; 36:3794–801. 29776750 10.1016/j.vaccine.2018.05.038 PMC6005168

[55] ClinicalTrials.gov. Safety and immunogenicity of Vi-DT typhoid conjugate vaccine (Bio Farma) in adults and children (phase I). Available at: https://clinicaltrials.gov/ct2/show/NCT03109600. Accessed 14 November 2018.

[56] van Damme P, Kafeja F, Anemona A, et al. Safety, immunogenicity and dose ranging of a new Vi-CRM197 conjugate vaccine against typhoid fever: randomized clinical testing in healthy adults. PLoS One 2011; 6:e25398. 21980445 10.1371/journal.pone.0025398 PMC3184126

[57] Bhutta ZA, Capeding MR, Bavdekar A, et al. Immunogenicity and safety of the Vi-CRM197 conjugate vaccine against typhoid fever in adults, children, and infants in South and Southeast Asia: results from two randomised, observer-blind, age de-escalation, phase 2 trials. Lancet Infect Dis 2014; 14:119–29. 24290843 10.1016/S1473-3099(13)70241-X

[58] Chinnasami B, Mangayarkarasi V, Prema A, Sadasivam K, Davis MJ Safety and immunogenicity of *Salmonella* Typhi Vi conjugate vaccine (PedaTyph) in children up to five years. Int J Sci Res Publ 2013; 3.

[59] Chinnasami B, Sadasivam K, Vivekanandhan A, Arunachalam P, Pasupathy S A study on longevity of immune response after vaccination with *Salmonella* Typhi Vi conjugate vaccine (PedaTyph) in children. J Clin Diagn Res 2015; 9:SC01–3. 10.7860/JCDR/2015/13302.5903 PMC4484117 26155525

[60] Mitra M, Shah N, Ghosh A, et al. Efficacy and safety of vi-tetanus toxoid conjugated typhoid vaccine (PedaTyph) in Indian children: school based cluster randomized study. Hum Vaccin Immunother 2016; 12:939–45. 26901576 10.1080/21645515.2015.1117715 PMC4962969

[61] Mohan VK, Varanasi V, Singh A, et al. Safety and immunogenicity of a Vi polysaccharide-tetanus toxoid conjugate vaccine (Typbar-TCV) in healthy infants, children, and adults in typhoid endemic areas: a multicenter, 2-cohort, open-label, double-blind, randomized controlled phase 3 study. Clin Infect Dis 2015; 61:393–402. 25870324 10.1093/cid/civ295

[62] Konadu EY, Lin FY, Hó VA, et al. Phase 1 and phase 2 studies of *Salmonella enterica* serovar Paratyphi A O-specific polysaccharide-tetanus toxoid conjugates in adults, teenagers, and 2- to 4-year-old children in Vietnam. Infect Immun 2000; 68:1529–34. 10678970 10.1128/iai.68.3.1529-1534.2000 PMC97311

[63] Lin FY, Ho VA, Khiem HB, et al. The efficacy of a *Salmonella* Typhi Vi conjugate vaccine in two-to-five-year-old children. N Engl J Med 2001; 344:1263–9. 11320385 10.1056/NEJM200104263441701

[64] Mai NL, Phan VB, Vo AH, et al Persistent efficacy of Vi conjugate vaccine against typhoid fever in young children. N Engl J Med 2003; 349:1390–1. 14523155 10.1056/NEJM200310023491423

[65] Meiring JE, Patel P, Patel P, Gordon MA. Typhoid conjugate vaccines: making vaccine history in Africa. Expert Rev Vaccines 2018; 17. 10.1080/14760584.2018.1496825 29972655

[66] Waddington CS, Darton TC, Woodward WE, Angus B, Levine MM, Pollard AJ Advancing the management and control of typhoid fever: a review of the historical role of human challenge studies. J Infect 2014; 68:405–18. 24491597 10.1016/j.jinf.2014.01.006

[67] World Medical Association Declaration of Helsinki: ethical principles for medical research involving human subjects. Available at: https://www.wma.net/policies-post/wma-declaration-of-helsinki-ethical-principles-for-medical-research-involving-human-subjects/. Accessed 4 January 2019. PMC2566407 11357217

[68] Bambery B, Selgelid M, Weijer C, Savulescu J, Pollard AJ Ethical criteria for human challenge studies in infectious diseases. Public Health Ethics 2016; 9:92–103. 29731811 10.1093/phe/phv026 PMC5926904

[69] Savulescu J Taking the plunge. New Scientist. 2001 Available at: https://www.newscientist.com/article/mg16922805-200-taking-the-plunge/. Accessed 4 January 2019. 12549456

